# Screening and Identifying m6A Regulators as an Independent Prognostic Biomarker in Pancreatic Cancer Based on The Cancer Genome Atlas Database

**DOI:** 10.1155/2021/5573628

**Published:** 2021-05-15

**Authors:** Bi Lin, Yangyang Pan, Dinglai Yu, Shengjie Dai, Hongwei Sun, Shengchuan Chen, Jie Zhang, Yukai Xiang, Chaohao Huang

**Affiliations:** ^1^Department of Anesthesiology, The First Affiliated Hospital of Wenzhou Medical University, Wenzhou, China; ^2^Department of Emergency Medicine, Yinzhou Second Hospital, Ningbo, Zhejiang, China; ^3^Department of Hepatological Surgery, The First Affiliated Hospital of Wenzhou Medical University, Wenzhou, China

## Abstract

**Background:**

Pancreatic cancer is one of the most malignant tumors of the digestive system, and its treatment has rarely progressed for the last two decades. Studies on m6A regulators for the past few years have seemingly provided a novel approach for malignant tumor therapy. m6A-related factors may be potential biomarkers and therapeutic targets. This research is focused on the gene characteristics and clinical values of m6A regulators in predicting prognosis in pancreatic cancer.

**Methods:**

In our study, we obtained gene expression profiles with copy number variation (CNV) data and clinical characteristic data of 186 patients with pancreatic cancer from The Cancer Genome Atlas (TCGA) portal. Then, we determined the alteration of m6a regulators and their correlation with clinicopathological features using the log-rank tests, Cox regression model, and chi-square test. Additionally, we validated the prognostic value of m6A regulators in the International Cancer Genome Consortium (ICGC).

**Results:**

The results suggested that pancreatic cancer patients with *ALKBH5* CNV were associated with worse overall survival and disease-free survival than those with diploid genes. Additionally, upregulation of the writer gene *ALKBH5* had a positive correlation with the activation of AKT pathways in the TCGA database.

**Conclusion:**

Our study not only demonstrated genetic characteristic changes of m6A-related genes in pancreatic cancer and found a strong relationship between the changes of *ALKBH5* and poor prognosis but also provided a novel therapeutic target for pancreatic cancer therapy.

## 1. Introduction

Pancreatic cancer, especially pancreatic ductal adenocarcinoma (PDAC), is one of the most malignant tumors of the digestive system, causing approximately 350,000 deaths worldwide every year [1]. By the year of 2030, PDAC would be the second leading cause of cancer-related mortality, surpassing lung cancer and colorectal cancer [2]. Although extensive studies on pancreatic cancer have been conducted over the past few decades, there is no substantial improvement in the prognosis of pancreatic cancer. The most optimal choice and therapeutic strategy for pancreatic cancer are still surgical resection combined with chemotherapy, but the unresectability rate is still high, and the 5-year overall survival (OS) rate remains <7%, accompanied with a continuous elevation of its incidence [1]. To date, there are still no reliable gene signatures for treatment effects and prognosis and for early detection and improved therapies. Therefore, there is an urgent need to develop a method to predict whether a patient will have a relatively long survival time and better prognosis, based on the characteristics of transcriptome sequencing and genome sequencing. Screening and uncovering potential biomarkers of tumor heterogeneity have become a focus in cancer research [3], and most of the candidate genes for therapeutic target are closely related to pathogenic gene variants or to their tumor tissue-specific antigens [4–6]. Many problems related to pancreatic cancer treatment remain to be solved, especially in the field of exploring the underlying molecular mechanisms of the tumorigenesis of pancreatic cancer and new therapeutic targets.

In the last few decades, chemical modifications of nucleobases have become a focus in controlling gene expression in cancers at different levels because of their subsequent regulation in protein translation and modulation of signaling pathways, especially N6-methyladenosine (m6A) modification. m6A has been considered the major type of normal endogenous modification on RNA molecules including mRNAs [6], miRNAs [7], and lncRNAs [8]. Additionally, m6A modification has been confirmed to be a reversible process dependent on multiple m6A regulatory enzymes, which are classified as “writers (WTAP, METTL3, and METTL14),” “erasers (FTO and ALKBH5),” and “readers (YTHDF1, YTHDF2, YTHDF3, YTHDC1, and YTHDC2)” [9].

The effects of m6A are mainly determined by m6A readers, writers, and erasers. The writer complex includes methylase enzymes, while the erasers downregulate the m6A level. Furthermore, the readers regulate the balance between writers and erasers, sequentially producing a functional signal [10]. Dysregulation of m6A results in multiple physiological homeostasis dysfunctions and affects the tumorigenesis and progression of most human malignancies through various mechanisms [11]. Therefore, m6A regulatory gene alternation also plays a vital role in multiple pathogenic mechanisms of human disease, especially in tumorigenesis [12]. However, the underlying mechanism of m6A regulatory genes is complex and involves multiple molecules and pathways [13]. Dysregulation of m6A regulatory genes in various cancers results in cancer cell EMT [14], apoptosis [15], and stem cell self-renewal [16], which are important in cancer progression. However, the relationship between m6A regulatory genes and pancreatic cancer is still unclear. Therefore, in our study, we obtained the RNA-sequencing (RNA-Seq) gene expression profiles and patients' clinical information of 186 patients with pancreatic adenocarcinoma (PAAD) from The Cancer Genome Atlas (TCGA). Among them, we evaluated the change profiles of 10 m6A regulatory genes in pancreatic cancer and the relationship between these changes and clinicopathologic features, including survival. Finally, an alteration of *ALKBH5* was identified, which could be considered a biomarker for prognosis or a therapeutic target for pancreatic cancer therapy.

## 2. Methods

### 2.1. Data Processing

From the TCGA database, we analyzed the copy number variation (CNV) data and pathology reports of a total of 186 patients with pancreatic cancer. The up-and-down regulation CNV was assessed using segmentation analysis and the GISTIC algorithm. Next, to explore the relationship between the clinicopathological significance of PAAD patients and m6A regulatory genes, the cohort of 186 pancreatic cancer patients was divided into two groups: “with mutation and/or CNVs of these m6A regulatory genes” and “without CNVs and mutation.” The mRNA expression data profiles of patients with pancreatic cancer were also acquired from the TCGA database, and then, the mRNA expression levels were processed with the log scale, exploring the association with CNVs.

Then, we acquired the clinical information and mRNA expression data profiles of patients with pancreatic cancer form Australia. After the data with ambiguous variables were excluded, the remaining 82 patients were processed by R software (R 4.0.3) with survival packages and GSEA software (GSEA 3.0).

### 2.2. Gene Set Enrichment Analysis (GSEA)

GSEA software (GSEA 3.0) was downloaded from the website (https://www.gsea-msigdb.org/gsea/index.jsp) [16]. Herein, according to the expression level of *ALKBH5*, GSEA divided the PAAD samples into two groups (*ALKBH5*-high group and *ALKBH5*-low group). Afterwards, the hallmark gene set “c2.cp.biocarta.v6.0.symbols.gmt” was set up in the GSEA to analyze 19726 genes involved in PAAD, and those with normalized *p* value < 0.05 were considered to be significantly enriched.

### 2.3. Statistical Analysis

SPSS 22.0 (IBM, Chicago, USA) and GraphPad Prism 7.0 (GraphPad Software, La Jolla, CA, USA) were applied to analyze the data. Our study adopted the *χ*^2^ test or Mann-Whitney *U* test to determine the relationship between m6A regulatory genes with different alternations and clinical characteristics of PAAD patients. The Kaplan-Meier curve and the log-rank test were adopted for analyzing the OS or/and disease-free survival (DFS) with m6A gene regulators. Then, a Cox proportional hazard regression model was used to analyze the relationship between m6A regulatory genes and clinicopathological characteristics of PAAD patients in terms of OS and DFS. A *p* value < 0.05 was considered to indicate a statistically significant difference.

## 3. Results

### 3.1. Mutations and CNVs of m6A Regulatory Genes in Patients with PAAD

**Figure 1:**
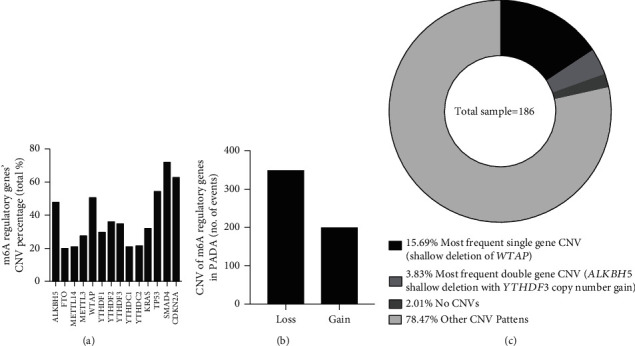
CNV characteristics of m6A regulatory genes in PAAD. (a) Percentage of PAAD samples with CNVs of m6A regulators, based on the data from TCGA. (b) Events of copy number gain or loss of m6A regulatory genes in PAAD samples. (c) The most common patterns of CNVs in m6A regulatory genes in the PAAD samples.

Within the TCGA database, only 19 independent samples were found to have mutations of m6A regulatory genes (Table 1), among the 186 cases based on the sequencing data; however, CNVs in ten m6A-related genes were observed in 177 PAAD samples based on the CNV data (Figure 1(a)). The results showed that the m6A “writer” gene *WTAP* had the highest frequency of CNV events (50.8%, 90/177) followed by *ALKBH5* (48.02%, 85/177), which is an m6A “eraser” gene. Furthermore, we also investigated the frequency of CNVs in *KRAS* (32.2%), *TP53* (54.2%), *SMAD4* (71.8%), and *CDKN2A* (62.7%) in this cohort.

Next, we determined the CNVs of the above ten m6A regulatory genes in the PAAD samples and found that the loss of the copy number was the most frequent CNV event (350/548) (Figure 1(b) and Table 2), which was the same as the CNV status in clear cell renal cell carcinoma (ccRCC) [17]. Among these CNVs, shallow deletion of *WTAP* ranked as the first in terms of the most frequent CNVs, and shallow deletion of both *ALKBH5* and *YTHDF3* was the most frequent cooccurring CNV, indicating the important roles these two genes play in the process of m6A RNA modification.

### 3.2. Alterations of m6A Regulatory Genes Are Associated with Clinicopathological and Molecular Characteristics

We also assessed the association between variations (CNV and/or mutation) of the m6A-related regulators and the clinicopathological features of patients with PAAD. Similar to the ccRCC samples, the results of this study showed a close correlation between alterations of m6A regulatory genes and higher Fuhrman Nuclear Grade (Table 3) in PAAD samples. On account of the fact that *KRAS*, *TP53*, *SMAD4*, and *CDKN2A* play important roles in the tumorigenesis of PAAD, we also assessed whether alterations of m6A regulatory genes were related to alterations of these four genes or not. As shown in Table 4, *KRAS*, *TP53*, *SMAD4*, and *CDKN2A* alterations in PAAD samples had a positive correlation with alterations of m6A regulatory genes as expected; meanwhile, one sample showed no alterations of m6A regulatory genes among the total 57 patients with KRAS CNV.

**Figure 2:**
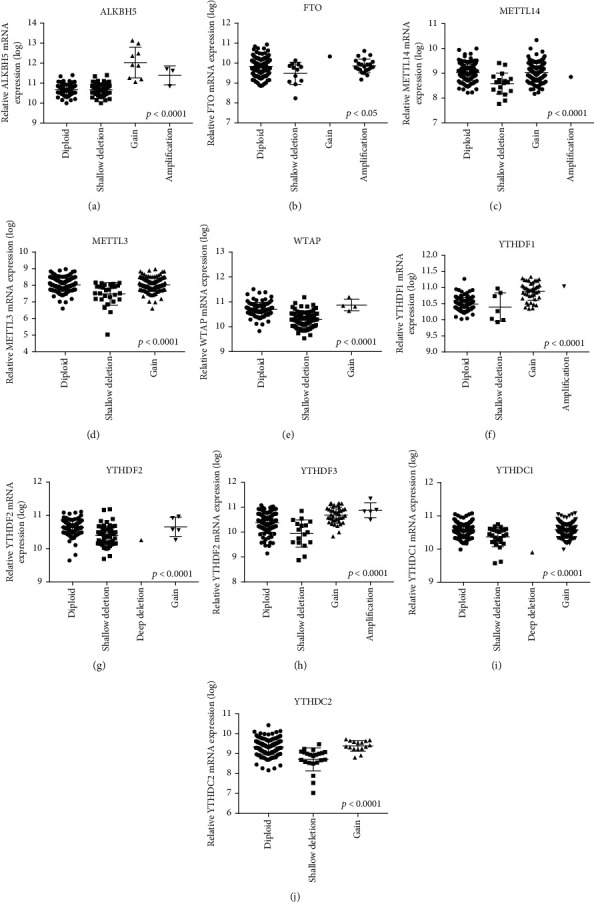
The relationship between different CNV patterns and mRNA expression levels of ten m6A regulatory genes in PAAD samples.

Furthermore, we also evaluated the association between the m6A regulatory genes and mRNA expression. The results revealed that the ubiquitous CNVs were associated with the mRNA expression levels of m6A-related genes in 177 PAAD samples. Among these genes, the copy number gains were positively associated with higher mRNA expression, whereas the shallow deletions or deep deletions were negatively associated with lower mRNA expression (Figure 2).

### 3.3. Identification of the Prognostic Value of m6A Regulatory Gene CNVs in Patients with PAAD

**Figure 3:**
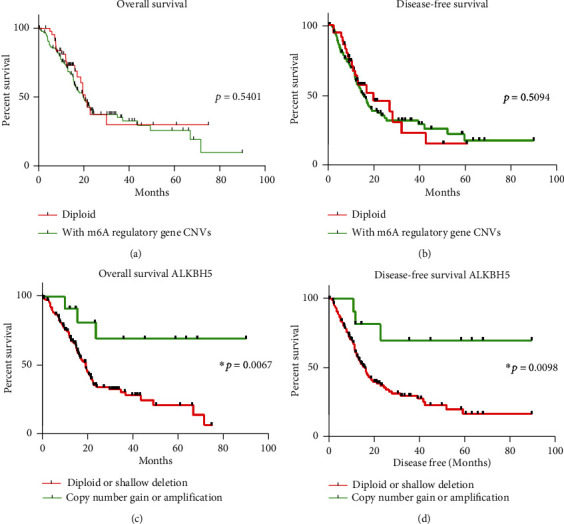
The overall survival of patients with PAAD with CNVs of m6A regulatory genes. (a, b) OS and DFS of patients with any of the CNVs of m6A regulatory genes or with diploid genes. (c, d) OS and DFS of patients with different CNV types of *ALKBH5*.

The CNV effects of m6A regulatory genes on the OS and DFS of patients with PAAD were evaluated. As shown in Figures 3(a) and 3(b), there was no correlation between m6A regulatory gene CNVs and OS/DFS among patients with PAAD. Furthermore, a separate analysis of the ten m6A regulatory genes revealed a significant difference between patients with PAAD and those with alterations of *ALKBH5* (one eraser gene of m6A). Copy number gain or amplification with *ALKBH5* showed better OS and DFS (Figures 3(c) and 3(d)); however, according to survival analysis of the CNVs of the other nine m6A-regulated genes, no significant differences were observed between the different separated subgroups (Figure S1). Additionally, *ALKBH5* was determined as an independent risk factor for OS and DFS, as shown in Table 5. Combined with the results presented above, we suggested that PAAD patients with upregulated *ALKBH5* mRNA expression have a better survival.

### 3.4. Enrichment Analysis of *ALKBH5* Gain of Function

**Figure 4:**
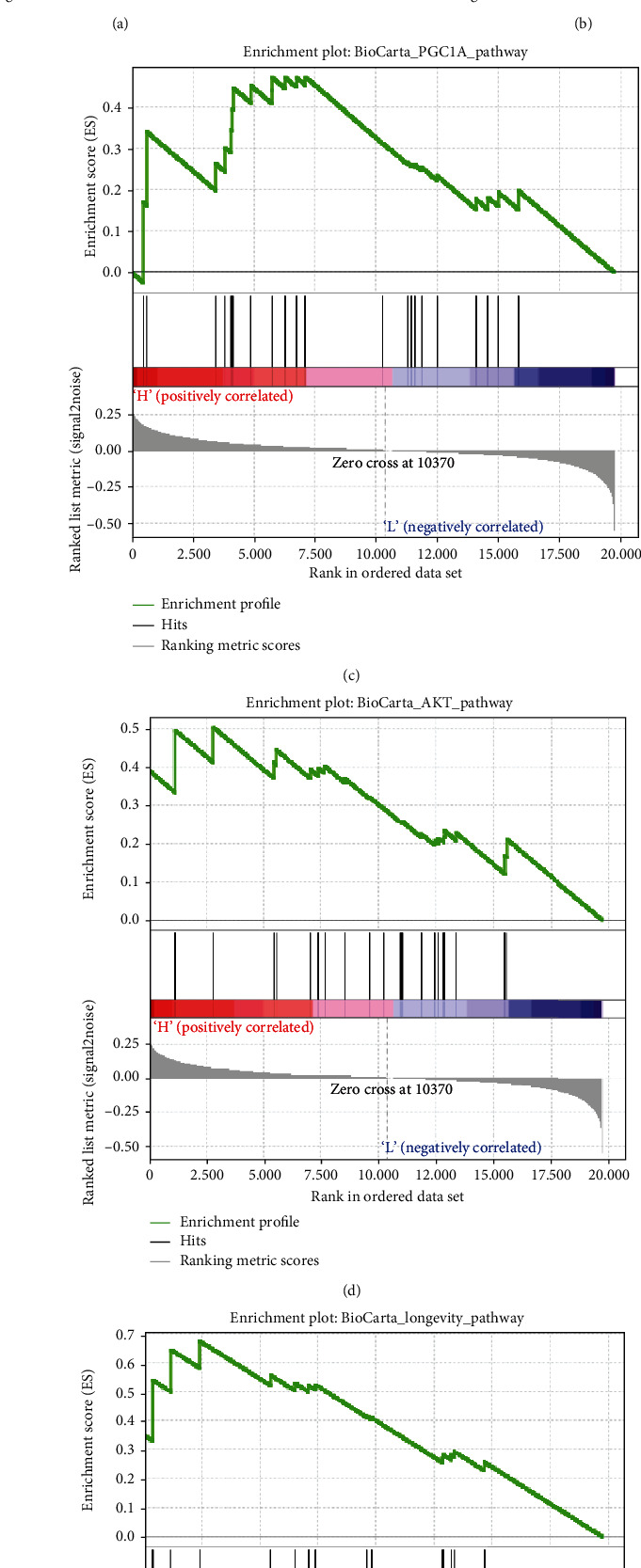
Functions of different expression levels of *ALKBH5* in TCGA. (a, b) OS and DFS of patients with different *ALKBH5* mRNA levels. Gene set enrichment plots of (c) PGC1A, (d) AKT signaling, and (e) longevity to *ALKBH5* mRNA levels in the PAAD samples.

To confirm the abovementioned conclusion of the relationship between upregulated *ALKBH5* expression and better and prolonged survival, we next evaluated the *ALKBH5* mRNA expression among patients with PAAD whose prognoses were affected by the *ALKBH5* mRNA level in Gene Expression Profiling and Interactive Analyses (GEPIA, http://gepia.cancer-pku.cn/index.html) [18]. As expected, patients with low *ALKBH5* mRNA expression had worse OS than those with high *ALKBH5* expression (Figure 4(a)). However, the *ALKBH5* mRNA expression level had no statistically significant association with DFS in patients with PAAD (Figure 4(b)). Considering *ALKBH5* as an “eraser” in the demethylation process, combining with the results of our study, we attempted to explore the dysregulation of *ALKBH5* in the pathogenesis of patients with PAAD. We examined the enriched gene sets in the TCGA data sample with different *ALKBH5* mRNA expression levels with GSEA. GSEA analysis showed that the differential expression of ALKBH5 was related to some key biological processes involving PGC1A, AKT, and longevity signaling pathways (Table 6 and Figures 4(c) and 4(d)), thus providing an indication of the underlying mechanism in the tumorigenesis of PAAD. Additionally, several studies have found that *ALKBH5* can participate in AKT signaling pathways, consistent with our assumption [19]. Further study is still needed to illustrate the potential effects of *ALKBH5* on the regulation of the downstream genes in pancreatic cancer.

### 3.5. Validating the Prognostic Value of m6A Regulators in ICGC Database

**Figure 5:**
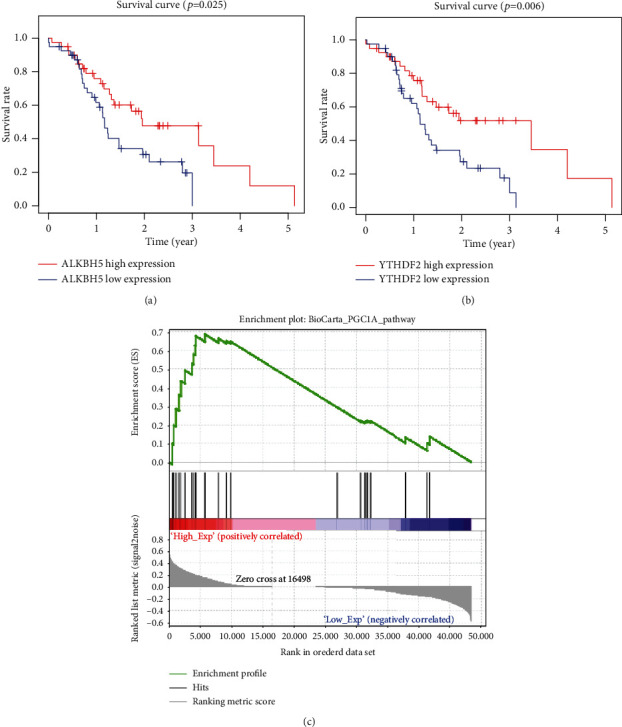
Functions of different expression levels of *ALKBH5* in ICGC. (a, b) OS of patients with different *ALKBH5* and *YTHDF2* mRNA levels. Gene set enrichment plots of (c) PGC1A to *ALKBH5* mRNA levels in the pancreatic cancer samples.

To confirm the prognostic value of alternative m6A regulators, we also analyze the m6A regulators in the ICGC database. The same in the TCGA database, patients with low *ALKBH5* mRNA expression had worse OS than those with high *ALKBH5* expression (Figure 5(a)). Moreover, patients with a higher YTHDF2 expression were observed to have a better overall survival (Figure 5(b)). Except for the above two genes, age, gender, and the expression of other m6A regulators had no significant differences with OS survival. However, GSEA analysis showed that the differential expression of *ALKBH5* in ICGC was only related to PGC1A signaling pathways (Table 7 and Figure 5(c)). The different results presented in the two databases might have resulted from the data offset because of the relatively small sample size.

## 4. Discussion

Pancreatic cancer is one of the most common malignancies of the digestive system, and progress in research related to its treatment has been slow. In recent decades, the discovery of m6A has increased our understanding of tumorigenesis regulation to a new level, helping us gain insight into the role of methylation and demethylation in tumor formation and progression [20]. Many studies have demonstrated that m6A alternation is one of the key factors in cancer management [21]. However, the role of m6A regulatory genes in pancreatic cancer remains unclear. Upon analysis of the different expressions or mutations of “readers,” “writers,” and “erasers” in different tissues, we found that the genes related to m6A regulation seem to be different in distant tumors. Therefore, in this study, we aimed to screen and uncover m6A regulatory factors closely related to clinicopathological significance and prognosis in pancreatic cancer. This study not only determined the value of m6A regulatory genes for pancreatic cancer prognosis but also proposed a novel therapeutic target for pancreatic cancer.

As a demethylase, ALKBH5 is involved in the mediation of methylation reversal. It has been reported that ALKBH5 is overexpressed in various cancers, including breast cancer [22], glioblastoma [23], ovarian cancer [24], and gastric cancer [25, 26]. Additionally, signaling associated with multiple cancers is dysregulated in PAAD development. We found that in patients with PAAD, a high *ALKBH5* mRNA expression level was associated with the activation of AKT signaling pathways, which participate in important cellular pathological processes in PAAD development [27], suggesting that the mRNAs of molecules in the AKT pathway may be the m6a modification target mediated by ALKBH5 [28]. Recently, a study has shown that ALKBH5 functions as an antitumor protein in pancreatic cancer progression [29]; in this paper, upregulated ALKBH5 sensitized pancreatic cancer to gemcitabine chemotherapy, and knockdown of ALKBH5 decreased pancreatic cancer cell invasion, migration, proliferation, metastasis, and tumorigenesis [29]. As in the case of colorectal cancer [30], *ALKBH5* showed obviously weaker mRNA expression in pancreatic cancer than in the normal tissue. However, in contrast to the case of rectal adenocarcinoma wherein high *ALKBH5* expression in tumor tissues was clearly associated with worse OS, *ALKBH5* expression in pancreatic cancer was found to be positively associated with OS in TCGA and ICGC, which was in accordance with the report by Cho et al. [31]. Compared with their study, we screened the 9 more m6A regulators and explored the CNV in the public database, evaluated the copy number variation (CNV) data of m6A on the OS and DFS of patients with PAAD, and further verified the prognostic value of ALKBH5 in pancreatic cancer.

We also evaluated the effect of m6A regulatory gene alterations on the survival of patients with PAAD. In line with the characteristics of genetic alterations of m6A-related genes, the eraser gene *ALKBH5* was the only gene among the ten regulators that was associated with the OS and DFS. This confirmed that “erasers” are the main regulators of m6A in PAAD. A better OS was observed in patients with eraser gene gain of function, making it clear that a decreased level of m6A plays a significant role in PAAD progression. However, we failed to obtain any significant results with regard to the relationship between the other nine m6A regulatory gene alterations and OS or DFS, possibly because of the limited number of patients. Direct detection of the m6A level and evaluation of its effect on PAAD survival in a new and larger cohort are needed to illustrate this contradictory phenomenon.

We also assessed the impact of m6A-related gene changes on prognosis, especially OS and DFS, in patients with PAAD. According to the genetic changes of m6A-related gene characteristics, only ALKBH5, an eraser gene, was associated with OS and DFS among the 10 regulatory genes. This confirms that erasers might be the predominant governors of m6A in PAAD. The patients with gained function of *ALKBH5* achieved better OS, indicating that decreased m6A levels may play an important role in the progression of PAAD. Since the different results of *YTHDF2* and *ALKBH5* were presented in the ICGC database, we were unable to derive any significant results on the relationship between the other nine m6A regulatory gene changes and OS or DFS, possibly due to the limited number of patients and database heterogeneity. To account for this paradox, m6A levels need to be directly detected, and their impact on PAAD survival should be evaluated in a new and larger cohort.

In conclusion, we screened alternations of ten m6A regulatory genes in the TCGA database of pancreatic cancer patients and identified that *ALKBH5* was the most valuable prognosis-related gene that may be associated with AKT signaling pathways. These findings revealed a novel molecular mechanism of PDAC tumorigenesis regulated by m6A modification and provided a new insight into the development of effective therapeutic strategies for the treatment of pancreatic cancer. Although we provided robust evidence for the prognostic value of the effect of ALKBH5 on pancreatic cancer, the underlying mechanism is not yet fully characterized. Thus, the effects of ALKBH5 clearly deserve further investigation.

## Figures and Tables

**Table 1:** Mutations of m6A regulatory genes in 186 PDAC patients.

PAAD sample ID	ALKBH5	FTO	METTL14	METTL3	WTAP	YTHDF1	YTHDF2	YTHDC1
TCGA-IB-7651	R327H	R473W						
TCGA-F2-A44G			R298H, X23_splice					
TCGA-2J-AABV				R471H				
TCGA-IB-7651				A191V				
TCGA-IB-7644					C161Y			
TCGA-HZ-A9TJ					X49_splice			
TCGA-IB-AAUS					K155E			
TCGA-IB-A5SQ						R404C		
TCGA-IB-7651								K565N, X374_splice, E224K, Q168H

**Table 2:** Different CNV patterns occur in PAAD samples (*n* = 177).

		Diploid	Deep deletion	Shallow deletion	Copy number gain	Amplification	CNV sum	Percentage
Eraser	ALKBH5	92		73	9	3	85	48.02%
	FTO	142		12	22	1	35	19.8%

Writers	METTL14	140		28	9		37	20.9%
	METTL3	128		19	29	1	49	27.7%
	WTAP	87		86	4		90	50.8%

Reader	YTHDF1	125		7	44	1	52	29.4%
	YTHDF2	113	1	58	5		64	36.2%
	YTHDF3	115		17	40	5	62	35%
	YTHDC1	141	1	26	9		36	20.3%
	YTHDC2	139		22	16		38	21.5%

	KRAS	120	1	14	36	6	57	32.2%
	TP53	81	2	86	8		96	54.2%
	SMAD4	50	25	95	7		127	71.8%
	CDKN2A	66	49	60	2		111	62.7%

**Table 3:** Clinical pathological parameters of PAAD patients with or without mutation/CNV of m6A regulatory genes^∗^.

		With mutation and/or CNV^∗^	Without mutation and CNV^∗^	*p*
Age	≤60	14	44	0.695
	>60	32	87	

Gender	Male	18	79	0.013^∗^
	Female	28	52	

Pathological stage	I	2	5	0.670
	II	9	15	
	III	34	107	
	IV	1	2	
	Discrepancy	0	1	
	N/A	0	1	

Historical grade	G1	13	17	0.035^∗^
	G2	24	71	
	G3	7	41	
	G4	1	1	
	Gx	1	1	

T stage	T1	9	12	0.328
	T2	35	111	
	T3	1	2	
	T4	1	3	
	Tx	0	3	
	NA	0	3	

M stage	M0	23	57	0.870
	M1	1	3	
	Mx	22	71	

N stage	N0	12	37	0.673
	N1	34	89	
	Nx	0	4	
	NA	0	1	

^∗^With mutation or CNV: cases have mutant or CNV or mutant+CNV, confirmed through TCGA database. Without mutant and CNV: cases with neither mutant nor CNV, confirmed through TCGA database. Ambiguous variables (Nx, Mx, N/A, discrepancy, and Gx) were excluded from chi-square test or nonparametric test. ^∗^*p* value < 0.005.

**Table 4:** Relationship between molecular characteristics and m6A regulatory gene alteration in PAAD patients.

		Without mutation or CNV^∗^	With mutation and CNV^∗^	*χ* ^2^	*p*
KRAS	Wt	45	75	25.671	*p* < 0.001^∗^
	Alteration	1	56		
SMAD4	Wt	32	18	52.346	*p* < 0.001^∗^
	Alteration	14	113		
TP53	Wt	42	39	51.936	*p* < 0.001^∗^
	Alteration	4	92		
CDKN2A	Wt	42	24	77.551	*p* < 0.001^∗^
	Alteration	4	107		

^∗^ *p* value < 0.005.

**Table 5:** Univariate and multivariate COX regression analyses of m6A regulatory genes for PAAD patients' overall survival (OS) and disease-free survival (DFS)^∗^.

	OS				DFS			
Variable	Univariate		Multivariate		Univariate	Multivariate	Univariate	Multivariate
	HR (95% CI)	*p*	HR	*p*	HR (95% CI)	*p*	HR	*p*
Age (>60 vs. ≤60)	1.383 (0.883-20165)	0.156	1.387 (0.854-2.253)	0.187	0.976 (0.622-1.533)	0.917	0.733 (0.446-1.205)	0.221
Gender (male vs. female)	1.192 (0.793-1.792)	0.398	1.561 (0.962-2.533)	0.071	1.137 (0.732-1.767)	0.568	1.506 (0.887-2.556)	0.129
Stage (I-II vs. III-IV)	1.165 (0.835-1.624)	0.369	1.168 (0.574-2.376)	0.669	2.120 (1.111-4.047)	0.023^∗^	1.162 (0.576-2.346)	0.675
M (M1 vs. M0)	1.050 (0.251-4.388)	0.947			0.936 (0.226-3.885)	0.928		
N (N1 vs. N0)	2.151 (1.281-3.612)	0.004^∗^	2.119 (1.184-3.793)	0.011^∗^	1.750 (1.065-2.875)	0.027^∗^	1.488 (0.844-2.623)	0.169
T (T3-T4 vs. T1-T2)	0.92 (0.250-2.513)	0.693	0.878 (0.258-2.987)	0.835	1.078 (0.339-3.431)	0.898	1.455 (0.419-5.056)	0.555
Grade (3-5 vs. 1-2)	1.496 (0.970-2.308)	0.069	1.146 (0.709-1.854)	0.578	1.740 (1.094-2.767)	0.019	1.424 (0.854-2.375)	0.175
KRAS (altered vs. diploid)	1.170 (0.75-1.825)	0.490	1.836 (1.058-3.185)	0.031^∗^	0.890 (0.547-1.449)	0.640	1.673 (0.869-3.219)	0.123
TP53 (altered vs. diploid)	1.242 (0.817-1.889)	0.311	1.140 (0.672-1.932)	0.628	0.986 (0.634-1.533)	0.950	0.986 (0.561-1.732)	0.961
SMAD4 (altered vs. diploid)	1.234 (0.766-1.987)	0.387	1.066 (0.590-1.926)	0.831	1.653 (0.976-2.800)	0.062	1.095 (0.568-2.110)	0.786
CDKN2A (altered vs. diploid)	1.307 (0.837-2.042)	0.238	0.950 (0.667-1.352)	0.774	1.480 (0.929-2.356)	0.099	0.915 (0.619-1.353)	0.657
WTAP (write loss vs. others)	1.325 (0.879-1.998)	0.179	1.291 (0.799-2.085)	0.298	1.245 (0.802-1.932)	0.329	1.255 (0.729-2.162)	0.412
Mettl3 (write loss vs. others)	0.918 (0.488-1.725)	0.790	0.648 (0.308-1.364)	0.253	1.269 (0.670-2.403)	0.464	0.869 (0.405-1.867)	0.720
Mettl14 (write loss vs. others)	1.199 (0.707-2.034)	0.501	1.378 (0.747-2.543)	0.305	1.639 (0.956-2.812)	0.073	1.970 (1.037-3.741)	0.038^∗^
FTO (eraser gain vs. others)	1.236 (0.684-2.234)	0.483	0.968 (0.483-1.942)	0.928	1.490 (0.799-2.778)	0.209	1.062 (0.499-2.263)	0.875
ALKBH5 (eraser gain vs. others)	0.229 (0.072-0.731)	0.013^∗^	0.287 (0.083-0.988)	0.048^∗^	0.199 (0.062-0.641)	0.007^∗^	0.201 (0.053-0.763)	0.018^∗^

^∗^Ambiguous variables (Nx, Mx, N/A, discrepancy, and Gx) were excluded. ^∗^*p* value < 0.005.

**Table 6:** Gene set enrichment of low ALKBH5 mRNA expression level in the PAAD cohort of TCGA.

GS details	Size	ES	NES	NOM *p* value
BioCarta_PGC1A_pathway	23	0.53	1.60	0.029^∗^
BioCarta_AKT_pathway	22	0.54	1.53	0.046^∗^
BioCarta_longevity_pathway	15	0.66	1.47	0.028^∗^

^∗^ *p* value < 0.005.

**Table 7:** Gene set enrichment of low ALKBH5 mRNA expression level in the PAAD cohort ICGC.

GS details	Size	ES	NES	NOM *p* value
BioCarta_PGC1A_pathway	23	0.68	1.50	0.031^∗^
BioCarta_AKT_pathway	21	0.67	1.39	0.091
BioCarta_longevity_pathway	15	0.59	1.22	0.214

^∗^ *p* value < 0.005.

## Data Availability

Publicly available datasets were analyzed in this study; these can be found in the International Cancer Genome Consortium (https://daco.icgc.org/) and The Cancer Genome Atlas (https://portal.gdc.cancer.gov/) by the cBioPortal platform and TCGA-Assembler, which are open to the public under some guidelines. The data analyzed during the current study are available from the corresponding author on reasonable request.

## Supplementary Materials

Supplementary Materials Figure S1: overall survival of patients with PAAD with CNVs of m6A regulatory genes excluding *ALKBH5*.
