# Supplementary figures and images for: Screening and Identifying m6A Regulators as an Independent Prognostic Biomarker in Pancreatic Cancer Based on The Cancer Genome Atlas Database
Source: Biomed Res Int. 2021 May 15;2021:5573628. doi: 10.1155/2021/5573628 (PMC8147537; doi:10.1155/2021/5573628)

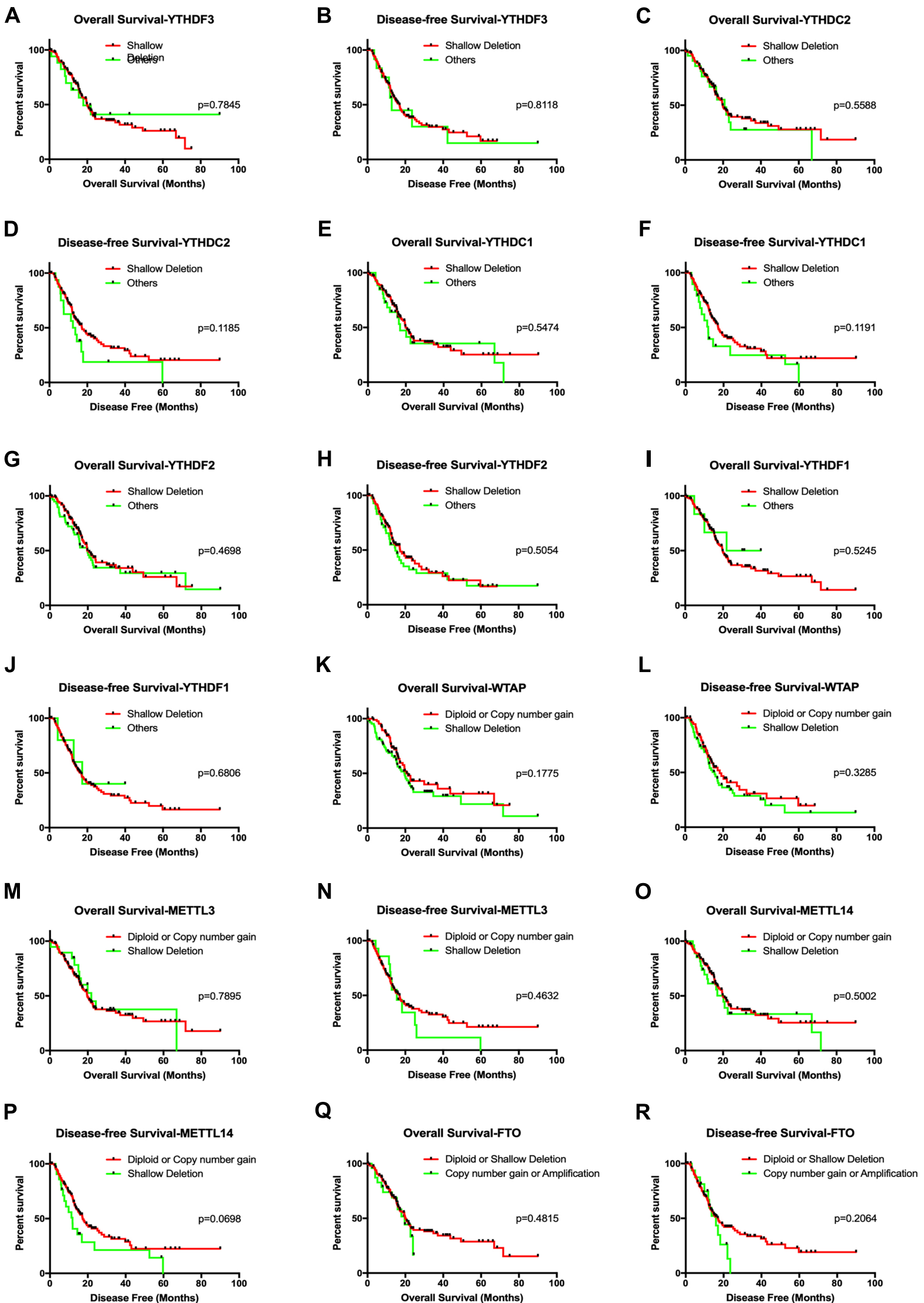

Supplement: Supplementary Materials — Figure S1: overall survival of patients with PAAD with CNVs of m6A regulatory genes excluding ALKBH5. [file 5573628.f1.pdf]
